# P-1728. Causal Machine Learning Algorithm improves the Sensitivity of LDBio Aspergillus ICT IgG/IgM Lateral Flow Assay in the Diagnosis of CPA in Nigeria

**DOI:** 10.1093/ofid/ofaf695.1899

**Published:** 2026-01-11

**Authors:** Oluwaseyi Jessy Balogun, Olawale O E Ajibola, Rita Okeoghene Oladele, Theophilus A Fashanu, Stephen Idowu Ogungbemi, Adeyinka A Davies, Nicholas Kayode Irurhe, Jean-Pierre Gangneux, David Denning

**Affiliations:** University of Lagos, Akoka Yaba, Lagos, Nigeria; University of Lagos, Akoka Yaba, Lagos, Nigeria; College of Medicine University of Lagos, Lagos, Lagos, Nigeria; University of Lagos, Akoka Yaba, Lagos, Nigeria; University of Lagos, Akoka Yaba, Lagos, Nigeria; Olabisi Onabanjo University Ago Iwoye, Ogun State, Sagamu, Ogun, Nigeria; College of Medicine University of Lagos, Lagos, Lagos, Nigeria; Rennes University hospital, Rennes, Rennes, Bretagne, France; The University of Manchester, Manchester, England, United Kingdom

## Abstract

**Background:**

*Aspergillus* LDBio ICT IgG/IgM lateral flow assay (LFA) is a cheap alternative to Bordier ELISA assay (BEA) for detection of fungal invasion in chronic pulmonary aspergillosis (CPA) patients in resource-limited settings. However, the sensitivity of LFA is inferior to BEA in the diagnosis of CPA and contributed to high misdiagnosis of CPA in low-income countries.

**Table 1:**
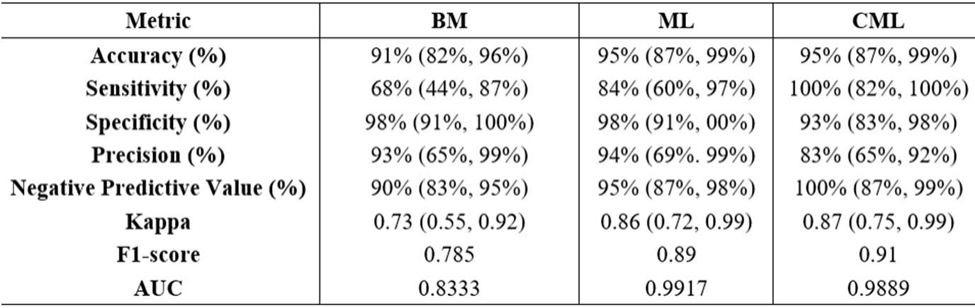
Performance of the Models on Testing data

**Figure 1:**
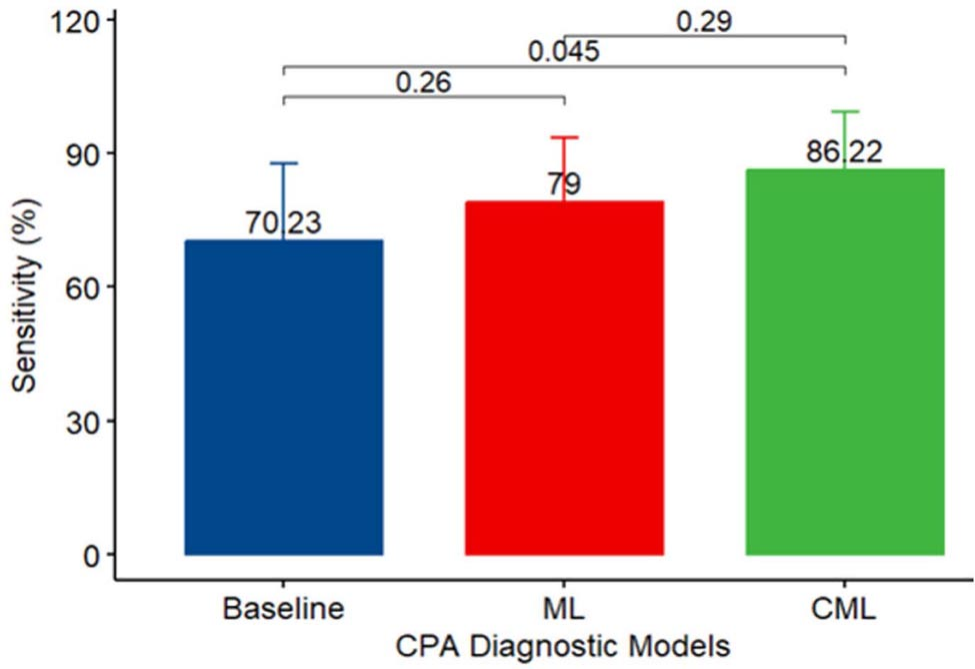
Comparison of Sensitivities across different Resampling

**Methods:**

We trained causal machine learning (CML) algorithm using symptoms, chest x-ray features, and IgG of LFA for diagnosis of CPA. This multi-center cross-sectional study included 386 pulmonary tuberculosis (PTB) patients, age 16 - 82 years, 214 (55%) female, 205 (53.1%) HIV positive, 138 (35.8%) post-TB, and 248 (64.2%) retreatments, with 97 (25.1%) having CPA. The BEA was positive in 98 (25.4%) cases while LFA was positive in 71 (18.4%) cases. CML is a dual logistic regression models (LR). The first model is a multivariable LR with symptoms and chest x-ray features as predictors. The second model is a simple LR having IgG of LFA as independent predictor. We also trained traditional multivariable LR (ML) with symptoms, chest x-ray features, and IgG of LFA as predictors of CPA. We used cross validation of 10-fold and partitioned data into 80:20 for training and testing samples. CML was compared to baseline model (BM) (physicians manually combining symptoms + chest x-ray findings + LFA) and traditional ML. CML, BM, and ML performance were assessed using the area under the receiver operating characteristic curve (AUC), accuracy, precision, sensitivity, specificity, kappa, and F1-score.

**Figure 2:**
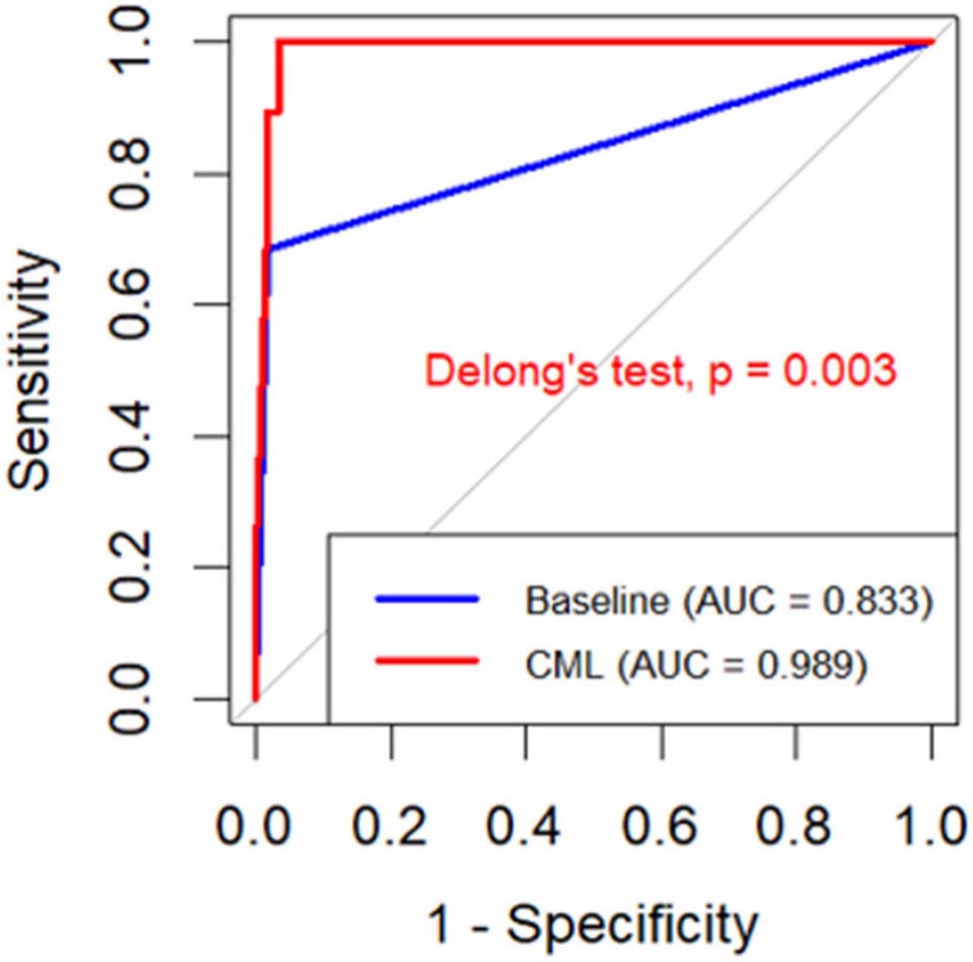
Comparison of Areas Under the Curve (AUCs)

**Results:**

The sensitivity of CML was 100% and superior to ML (84%) and BM (68%). CML [Accuracy = 95%, AUC = 0.989] was substantially higher than BM [Accuracy = 91%, AUC = 0.833]. The Cohen' s Kappa of CML (0.91) surpassed BM (0.79). The specificities > 90% for BM, ML, and CML.

**Conclusion:**

CML algorithm outperformed BM and reduced misdiagnosis of CPA among PTB patients. CML algorithms will aid physicians in optimal diagnosis of CPA in resource-challenge environments.

**Disclosures:**

Olawale O.E. Ajibola, N/A, Ph.D, University of Lagos: Grant/Research Support

